# The role of fuzzy logic in malaria transmission^☆^

**DOI:** 10.1016/j.nmni.2025.101694

**Published:** 2025-12-31

**Authors:** Gizachew Tirite Gellow, D.L. Suthar

**Affiliations:** a Department of Mathematics, Debre Tabor University, P.O. Box 272, Debre Tabor, Ethiopia; b Department of Mathematics, Wollo University, P.O. Box 1145, Dessie, Ethiopia; c Department of Mathematics, Saveetha School of Engineering, Thandalam 600124, Chennai, India

**Keywords:** Algorithm, Inference, Defuzzification, Fuzziness, Logic

## Abstract

Fuzzy logic is typically used to create fuzzy rules that are simple enough for people to understand, and it has been utilized in numerous industries where uncertainty is important. One outstanding example of ambiguity and uncertainty is medical diagnosis. We employ fuzzy logic, which builds a logic as the foundation for inference rules using the entire real number range between zero and one. Three things set it apart: it makes use of linguistic variables; it represents relationships between variables using conditional statements; and for complex relations, it employs fuzzy algorithms.

## 1 Introduction

Recent studies have highlighted the complexities of malaria transmission through various mathematical modeling approaches. [1] conducted an in-depth analysis of a ten-compartmental mathematical model, shedding light on the dynamics of malaria spread and the impact of interventions on transmission rates. Expanding on this, [2] presented a mathematical model that offers further insights into the epidemiology of malaria, emphasizing the importance of precise modeling in understanding disease dynamics. Such models are critical in informing public health strategies and guiding effective malaria control measures.

The study of fuzzy structures, or structures including fuzziness, is known as fuzzy mathematics. These are mathematical structures that, at some points, substitute a bigger structure of degrees for the two classical truth values, 0&1 (see [3], [4]). A finite set, an arbitrary lattice, and some sort of algebra are common alternatives to the real unit interval [0,1], which is frequently used as the system of degrees [5]. The purpose of the degrees is to offer a fuzzy mathematical structure greater flexibility than what the two truth degrees do for the matching classical mathematical structure [3], [6]. The fuzzy set ψ of X has unique function linked to it, unlike a crisp set, if X depicts the Universe of dialogue and its elements are denoted by x. $\mu_{\psi }:\left(x\right)\to \left[0,1\right]$ is the definition of a membership function. denotes the fuzzy set in which

(1) $$\mu_{\psi }\left(x\right)=\left\{\begin{array}{cc} 1 & \;\text{if}x\text{is totally in}A\;\\ 0 & \text{if}x\text{is not in}A\;\\ 0<\mu_{\psi }\left(x\right)<1 & \;\text{if}x\text{is partially in}\psi \end{array}\right)$$

that is $\mu_{\psi }\left(x\right)$ expresses to which the value x belongs to the fuzzy set ψ. The value 0 denotes absolute non-membership, while the number 1 denotes absolute membership. A fuzzy membership function thus indicates the degree of belonging to a component x of the universe of discourse X. Each element of X is mapped to a membership grade within 0&1 using a range of fuzzy membership functions, including polynomial curves, sigmoid functions, generalized bell-shaped, triangular, trapezoidal, and Gaussian curves. The simplest and most widely used of them is the triangle, which consists of three points. The memberships at the two extremities are 0 and the apex is 1. However, the trapezoid member function offers further details. It is essentially a truncated triangle curve with a flat top. With four points, it is comparable to the triangle. According to [4], [6], [7], it has an interval where the membership value is 1. This study will make use of the widely utilized triangular membership function. Fuzzy logic is characterized by three attributes. To express the relations between variables, conditional statements are used as the first feature [8]. Finally, fuzzy algorithms are used for complex relations. Here is how to calculate the triangular membership function:

(2) $$\mu_{\psi }\left(x\right)=\left\{\begin{array}{cc} 0 & ifx\leq a\\ \frac{x-a}{c-a} & ifx\in \left[a,c\right]\\ \frac{b-x}{c-b} & ifx\in \left[b,c\right]\\ 0 & ifx\geq c, \end{array}\right)$$

where medical professionals have defined a,b&c. Since fuzzy logic models may translate numerical values into linguistic variables that end users of the system can easily understand, one of their most notable features is their ability to represent linguistic variables. Typically, fuzzy logic is employed to construct human-readable fuzzy rules [9], [10], [11]. Fuzzy variables are hence frequently referred to as linguistic variables. In this work, we employ the following linguistic variables: mild, moderate, severe, and very severe for both the fuzzy model’s input and output parameters. The fuzzy system’s primary output, fuzzy if-then rules, would be established using those language variables: expressed typically as follows: if x is A, then y is B, where A&B and x&y are linguistic values, as determined by their fuzzy sets, and x&y are linguistic variables Called the antecedent, the initial portion of the rule may be composed of more than one part joined by the operators AND or OR. Also containing several outputs, the later section is referred to as the consequent.

Our paper aims to explore the use of fuzzy logic in predicting malaria transmission patterns and evaluate the economic viability of fuzzy logic-based methods in malaria control. We focus on the implementation of these methods in low-cost settings, highlighting successful examples and discussing the observed outcomes and benefits in these case studies including the work by [12]. Current malaria diagnostic tools, such as rapid diagnostic tests (RDTs) and microscopy, have notable limitations. RDTs can yield false positives or negatives, and their effectiveness varies depending on parasite species and parasitemia levels. While microscopy is considered the gold standard, it requires trained personnel, is time-consuming, and is less sensitive at low parasite densities. These issues can lead to misdiagnosis and improper treatment, which adversely affects patient care and public health efforts. By addressing these limitations, our study aims to provide a comprehensive evaluation of fuzzy logic-based methods in malaria control. We emphasize the practical application of these methods in resource-limited settings and assess their potential in enhancing diagnostic accuracy and efficiency. The paper also includes case studies to illustrate the observed outcomes and benefits, providing valuable insights into the economic viability and overall effectiveness of fuzzy logic approaches in malaria management.

## 2 Fuzzy control logic system (FCLS)

According to [9], [13], degrees of membership — as opposed to crisp membership — are the basis for the mathematical concepts that determine FCLS-based knowledge representation. In order to tackle the ambiguity, imprecision, and labor-intensive nature of standard laboratory malaria diagnosis, it helps medical professionals and researchers study the disease. Based on the data provided, it produces correct results [14]. According to [7], the opinions of domain experts on a range of study-related issues determine the effectiveness of a fuzzy expertise system. Fuzzy logic is used in the rule-based FCLS system in place of Boolean logic. Architecture of FCLS is given in Fig. 1.

We created an FCLS in this paper. Based on human understanding, medical diagnosis, and clinical observations, FCLS was designed. The system’s objective is to provide physicians, researchers, and other healthcare workers in malaria-endemic areas with an opportunity for decision support. Systems utilizing human intelligence are now essential to the healthcare industry’s survival and growth. Human expert systems will inevitably be applied in medical applications. A significant number of tropical illness expert systems have been developed by [15], [16]. An expert system on tropical diseases was created by [11] to help paramedical workers with training and the identification of numerous frequent diseases seen in their clinics. [3] built an expert system on endemic tropical diseases in another study. Many expert systems are available for the diagnosis and management of certain medical conditions. [10], [11] suggested an HMES for treating tropical diseases, which was intended to help doctors identify symptoms associated with a specific tropical disease, predict the likelihood of the condition, and recommend potential treatments based on the HMES diagnosis. The two knowledge structures used by the HMES diagnostic are symptoms and disease. Utilizing a forward chaining method, the HMES diagnosis inference engine searches the knowledge base for disease symptoms and associated treatments that match the patient’s query.

**Fig. 1:**
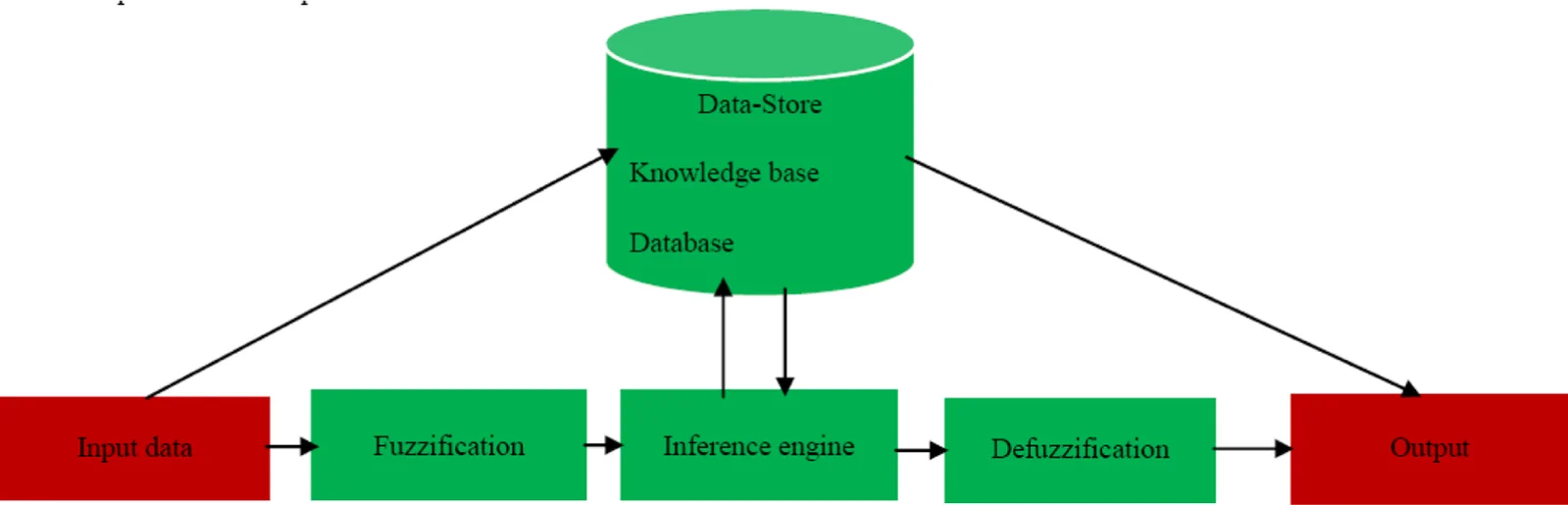
Architectural Structure Of FCLS.

To enhance making choices for malaria academics, institutions, and other medical professionals in malaria-endemic places globally, [11] developed a specialist system for malaria ecological diagnostics. The impetus for this project came from the dearth of available malaria control methods and the necessity for novel malaria prevention tactics. Several articles have effectively outlined the advantages and difficulties of applying clinical decision support systems. [3], [17] Developed a Clinical Decision Support System for Malaria Diagnosis: A Case Study Comparing Two Soft Computing Techniques. Similar to [11], an experimental comparison between fuzzy logic and the analytic hierarchy method was built for medical decision support systems. The methods used in control theory to create conventional controllers are based on mathematical representations of the open-loop process, or system, that has to be regulated. Within a fuzzy logic controller, a set of linguistic description rules grounded in expert knowledge characterize the dynamic behavior of a fuzzy system. The process of creating fuzzy logic control systems often includes the following steps: developing fuzzy mapping and rules; building algorithms; creating knowledge-based systems (fuzzification); inferring; and defuzzifying.

### 2.1 Designing algorithm

Fuzzy control rules are a useful way to represent domain knowledge and control policy. Multiple language factors may also be involved in the antecedent and conclusion of these rules. The system will be referred to as a multi-input-multi-output fuzzy system in this situation. The fuzzy logic controller can be expressed in a manner akin to the traditional control law, U(k)=F(e(k),e(k−1),…..,e(k−τ),U(k−1),…,U(k−τ)), where a fuzzy rule-base describes the function F. That does not, however, imply that the fuzzy logic controller is a type of difference equation or transfer function. Given that it is unrealistic to anticipate significant linguistic assertions for e(k−3),e(k−4),…,e(k−τ), fuzzy logic control’s knowledge-based nature necessitates a restricted application of the error and control u’s past values. A traditional fuzzy logic control describes the connection between the changes in the control, the mistake e(k), and the modification of Δe=e(k)−e(k−1). As an alternative, this control rule can be represented as ΔU(k)=F(e(k),Δe(k)), which is a broad fuzzy logic control expression. The true output of the regulator U(k) is obtained by updating the prior value of U(k−1).

(3) ΔU(k)U(k)=U(k−1)+ΔU(k).

It was initially put forth by Mamdani and Assilian in 1975 and is referred to as the Mamdani-type fuzzy logic control. An example rule base for a simple fuzzy logic control system that applies the previously mentioned control law is as follows:

$$R_{1}:If\;e\;is\;positive\;\&\;\Delta e\;is\;near\;zero,\;then\;\Delta U\;is\;positive,$$

$$R_{2}:If\;e\;is\;negative\;\&\;\Delta e\;is\;near\;zero,\;then\;\Delta U\;is\;negative,$$

$$R_{3}:If\;e\;is\;near\;zero\;\&\;\Delta e\;is\;near\;zero,\;then\;\Delta U\;is\;near\;zero,$$

$$R_{4}:If\;e\;is\;near\;zero\;\&\;\Delta e\;is\;positive,\;then\;\Delta U\;is\;positive,$$

$$R_{5}:If\;e\;is\;near\;zero\;\&\;\Delta e\;is\;negative,\;then\;\Delta U\;is\;negative$$

Furnishing crisp inputs with fuzzy sets is a necessary step in fuzzy rule-based systems. Defuzzification is necessary in order to achieve a crisp value because a fuzzy system will always provide a fuzzy set. The effectiveness of fuzzy logic management systems is determined by the judgments of the domain specialists on various study-related concerns. The prescribed algorithm for the fuzzy control diagnostic process of malaria, as shown in Fig. 1, is:

step 1 *Include the symptoms and indicators of the patient’s complaints in the database. m=the total amount of signs and symptoms.*

step 2 *Look up* d *in the information base for the disease. There is a list of its signs and symptoms.*

step 3 *Find the corresponding severity degree by using the following weighing factors: mild=1, moderate=2, severe=3, very severe= 4.*

step 4 *Apply fuzzy rules.*

step 5 *Map fuzzy inputs into the appropriate weighing factors for determining the degree of membership.*

step 6 *Determine the non-minimum numbers evaluation rule.*

step 7 *Find the rules* R*’s firing strength.*

step 8 *By figuring out each rule’s none zero minimum value, you may determine the level of truth, or* R*.*

step 9 *Determine the disease’s severity.*

step 10 *Output fuzzy diagnosis*

### 2.2 The knowledge based system(fuzzification)

An important component of intelligent systems’ functionality is knowledge. A knowledge base is a special kind of database that may be used to collect, organize, disseminate, search, and utilize information. It is utilized in knowledge management. The expertise of domain experts in tropical medicine is represented succinctly and structured in fuzzy control logic systems for the treatment of malaria. Because the knowledge base contains precise information and guidelines regarding the problem, and the inference engine grants reasoning power, the expert system is capable of drawing conclusions. Structured knowledge consists of facts, instructions, and cases related to tropical diseases that are generally acknowledged by experts in the discipline of tropical medicine. In this work, malaria is taken into consideration as a recognized tropical illness. The first step in developing a system for experts based on fuzzy logic is to build sets of fuzzy values for the parameters.

The four language variables mild, moderate, severe, and extremely severe were used to define the input and output parameters chosen for this work based on the knowledge of domain experts. Eqs. (4) through (7) below illustrate this. Table 1 displays the range of fuzzy values for each language.

Eqs. (4) to (7) below express the functions that are used to alter the raw data before fuzzification begins. As indicated in Eqs. (4) to (7) below, linguistic variables are assessed using a triangular membership function during the process and are accompanied by corresponding degrees of membership ranging from 0 to 1. Both literature and knowledgeable medical professionals in the field of tropical medicine are used to determine these formulations.

(4) $$\mu_{mild}\left(X\right)=\left\{\begin{array}{cc} 0 & \text{if}\;x\leq 0.1\\ \frac{x-0.1}{0.2} & \;if\;0.1\leq x\leq 0.3\\ \frac{0.2-x}{0.1} & \;if\;0.2\leq x\leq 0.3\\ 0 & if\;x\geq 0.2 \end{array}\right)$$

(5) $$\mu_{Moderate}\left(X\right)=\left\{\begin{array}{cc} 0 & if\;x\leq 0.3\\ \frac{x-0.3}{0.3} & if\;0.3\leq x\leq 0.6\\ \frac{0.45-x}{0.15} & if\;0.45\leq x\leq 0.6\\ 0 & if\;x\geq 0.45 \end{array}\right)$$

(6) $$\mu_{severe}\left(X\right)=\left\{\begin{array}{cc} 0 & if\;x\leq 0.5\\ \frac{x-0.6}{0.2} & if\;0.6\leq x\leq 0.8\\ \frac{0.7-x}{0.1} & if\;0.7\leq x\leq 0.8\\ 0 & if\;x\geq 0.7 \end{array}\right)$$

(7) $$\mu_{verysevere}\left(X\right)=\left\{\begin{array}{cc} 0 & if\;x\leq 0.8\\ \frac{x-0.1}{0.2} & if\;0.8\leq x\leq 1.0\\ \frac{0.2-x}{0.1} & if\;0.9\leq x\leq 1.0\\ 0 & if\;x\geq 1.0 \end{array}\right)$$

Fuzzy rule formulation is the next stage of the fuzzification process. With domain expert support, the fuzzy rules for this paper were built. Composition theory is used in the formulation of numerous fuzzy rules in the knowledge-based fuzzy expert system of malaria transmission. Expert doctors selected only the rules that were valid. A sample of fuzzy rules for malaria is presented in Table 4 below.

**Table 1:** Range of fuzzy values.

Linguistic variables	Fuzzy values
Mild (Low)	0.1≤ x <0.3
Moderate (Average)	0.3≤ x <0.6
Severe (High)	0.6≤ x <0.8
Very Severe(very high)	0.8≤ x ≤1.0

### 2.3 Inference system

Inference is the process of making conclusions based on available information. Fuzzy inference is the process of applying fuzzy set theory to map an input to an output. Most of the making choices output is processed by the inference engine using the rules present in the rule base. The engine for fuzzy inference makes deductions based on the knowledge base’s rules. The fuzzy logic controller of the system for the administration of the mosquito transmission inference engine uses a forward chaining strategy to search the knowledge for illness indicators. The inference mechanism searches for the membership values in each rule’s condition. To ascertain the degree of membership, fuzzy inputs are mapped into the corresponding weighting factors and linguistic variables. To determine a rule’s firing strength or degree of fulfillment, utilize the aggregate operator. We have chosen to use fuzzy logical AND in this study in order to assess the rules’ combined firing strength. Though their definitions tend to differ, fuzzy logical operators like AND, OR, &, and NOT are typically used in practice to combine several antecedents of fuzzy rules sets. Essentially, AND employs the lowest weight among all antecedents, whereas OR employs the highest value. Another operator is NOT, which returns the complementary function by subtracting a membership function from 1.

The preceding and following of a rule are its IF and THEN clauses, respectively. To combine the antecedent sections of the rules for the purposes of this research, utilize the AND operator. Using the AND operator to evaluate the non-zero minimum values, each rule’s degree of truth (R) is found. The inference engine evaluates each rule in the regulation baseline and weighs the consequences of each applicable (fired) rule before producing a single fuzzy set. The Root Sum Square inference engine method is used in this work. Equation gives the root sum square.

(8) $$\sqrt{\Sigma R^{2}}=\sqrt{R_{1}^{2}+R_{2}^{2}+R_{3}^{2}+\cdots +R_{n}^{2}}$$

If R= value of firing rule, then $R_{1}^{2}+R_{2}^{2}+R_{3}^{2}+\cdots +R_{n}^{2}$ are strength values (truth values) of several rules that share the same conclusion. The fuzzy center of the composites region is calculated using a root sum square technique, which also adjusts the functions to match their respective magnitudes and integrates the effects of all applicable rules. Although this approach has greater mathematical complexity than other approaches, it was chosen for this paper because it provides the best weighted influence on all firing rules. Examples of rule-based assessments for a few patient numbers, with the remaining numbers evaluating similarly. The best method for deriving data from the established rules was determined to be the Root Sum Square of Drawing Inference.

### 2.4 Defuzzification system

The output of the inference engine is transformed into clear output using the defuzzification process. This is due to the fact that most medical applications require precise values, while most inference engines produce fuzzy sets as their output. A fuzzy set is fed into the defuzzification process, which yields a crisp output. The process of making control outputs or conclusions out of a combination of fuzzy rules, output membership functions, and input that is still unclear or fuzzy is known as fuzzy inference. A defuzzification process needs to be established prior to the utilization of those conclusions in real-world scenarios. Returning the fuzzy output to a control target as conventional output is the aim of the defuzzification procedure. Remember that the ambiguous result or conclusion is still a language variable that needs to be defuzzified in order to become a clear variable.

There are six common defuzzification techniques: Poor c-means (FCM), the bisector of area (BOA), the mean of maximum (MOM), the largest of maximum (LOM)& C-means fuzzy (COG)... Three defuzzification methods are commonly employed: the center of the force of gravity approach, the mean of the highest method, and the height method (HM). With the MOM defuzzification technique, the mean of the highest-degree fuzzy outputs or conclusions is computed. A drawback of the MOM method is that it manages the output membership function’s points with the greatest degrees only, not the function’s entire form. The same outcome will be obtained using this procedure for membership functions with different forms but comparable highest degrees. This defuzzification technique is useful only in the case where the final membership function is a combination of union results of symmetrical functions [18]. This process is divided into two phases. First, we can convert the corresponding membership function $F_{i}$ into a crisp subsequent $x=f_{i}$, where $f_{i}$ denotes the center of gravity of $F_{i}$. The COG technique, which can be expressed as

(9) $$x=\frac{\Sigma W_{i}f_{i}}{\Sigma W_{i}}$$

where $W_{i}$ represents how well the input data matches the ith rule. This method’s simplicity is one of its benefits. As a result, this defuzzification technique is widely used in neuro-fuzzy models to simplify computational complexity. In real-world applications, the Center of Gravity Method is the most extensively used defuzzification technique. [6] was the person who created this method. This thesis uses this approach because it makes intuitive sense and is easily computed. This technique is comparable to the physics formula Advanced Fuzzy Logic Technologies in Industrial Applications for determining the center of gravity. The most precise value of the fuzzy quantity is calculated as the weighted average of the membership function or the center of gravity of the region bordered by the membership function curve. he most popular method, known as the Center-of-gravity or centroid method, can be used to represent the COG output. For this paper, we used this method because, when applied, the defuzzified values tend to move smoothly around the fuzzy output region, providing a more accurate representation of a fuzzy set of any shape. To get around this drawback, the center-of-gravity frequently employs discrete variables, as demonstrated in equation. This use a weighted average of the fuzzy set’s centers rather than integration in (10). Averaging is done using the center-of-gravity method. Here is the formula for the center of gravity:

(10) $$The\;center\;of\;gravity\;\left(X\right)=\frac{\Sigma \mu_{X}\left(x_{i}\right)x_{i}}{\Sigma \mu_{X}\left(x_{i}\right)},$$

where the membership function’s center is $x_{i}$, and the membership value is $\mu_{X}\left(x_{i}\right)$. As shown in Fig. 2, this graphic representation illustrates the concept of the center of gravity.

### 2.5 Fuzzy mapping & implication rules

Fuzzy control rules can be applied to any applicable field of application by a specialist. An IFTHEN sequence is used to express the fuzzy rule in a closed-loop control system. This sequence leads to algorithms that specify what output or action should be performed based on the observed data at that moment. These algorithms incorporate both input and feedback. Human knowledge and experience are the foundation for designing or creating a set of fuzzy rules, and their applicability varies depending on the situation. A ambiguous IF−THEN rule associates a condition stated in terms of linguistic variables and fuzzy sets with an outcome or a conclusion. The result or conclusion might be given as a linguistic variable in the THEN section.

The IF element is mostly used to collect information utilizing the elastic conditions. The fuzzy inference system makes extensive use of this IF−THEN rule to determine how closely the input data meets a rule’s condition. There are two types of uncertain control rules that are typically employed for most practical applications. Fuzzy implication rules are another name for fuzzy mapping rules. To provide an operational mapping among the data being provided and the output, fuzzy mapping rules make use of linguistic variables. Fuzzy graphs, which show how fuzzy input as well as fuzzy output are connected, serve as the foundation for fuzzy mapping rules. Real-world applications can present challenges in deriving particular relationships between input and output, or even in cases where relationships are developed, they can be quite complex.

In those circumstances, fuzzy mapping rules are an excellent option. Since each fuzzy mapping rule only approximates a small subset of the function’s elements, a set of fuzzy mapping rules should be used to approximate the full function. Fuzzy mapping rules act similarly to human intuition or insight. There should be more fuzzy rules used and more short parts for the input and output. The fuzzy implication rule characterizes an input–output general logic implication relationship. The restricted interpretation of fuzzy logic forms the basis of a fuzzy implication rule. Classical two-valued logic and multiple-valued logic are related to fuzzy implication rules. Creating fuzzy rules is the next stage in the fuzzification process. Experts in the field helped construct the fuzzy rules for this paper. Combination theory was used to generate a large number of fuzzy rules that formed the knowledge foundation for the fuzzy-logic control systems used in malaria management. Malaria is considered mild if the following conditions are met: bodily weakness is mild, dizziness is severe, appetite loss is mild, MP is mild, fever is mild, headache is mild, nausea is mild, vomiting is modest, jaundice is mild, and enlarged liver is mild.

Law 1 *“If the following conditions are met: mild fever, mild headache, mild nausea, moderate vomiting, mild jaundice, mild enlarged liver, mild joint pain, mild body weakness, severe disorientation, mild appetite loss, mild MP, and mild malaria”.*

Law 2 *“Malaria is moderate if the following conditions are met: body weakness is severe, dizziness is very severe, lack of appetite is severe, MP is moderate, fever is moderate, headache is minor, nausea is mild, vomiting is mild, jaundice is mild, and enlarged liver is modest”.*

Law 3 *“If the following criteria are met: fever = severe; headache = moderate; nausea = mild; vomiting = mild; jaundice = mild and enlarged liver = mild; joint pain = mild; bodily weakness = medium; dizziness = severe; appetite loss = severe and MP = moderate; then malaria = severe”.*

Law 4 *“If the following conditions are met: fever = very severe; headache = mild; nausea = mild; vomiting = mild; jaundice = mild; enlarged liver = mild; joint pain = severe; bodily weakness = severe; dizziness = mild; appetite loss = mild; MP = severe; THEN malaria = extremely”.*

Law 5 *“Malaria is considered moderate if the following conditions are met: physical weakness is moderate, dizziness is moderate, lack of appetite is severe, MP is moderate, fever is moderate, headache is minor, nausea is mild, vomiting is moderate, jaundice is mild, and an enlarged liver is mild”.*

Law 6 *“If the following conditions are met: mild fever, moderate headache, moderate nausea, mild vomiting, slight jaundice, mild enlarged liver, mild joint pain, mild bodily weakness, moderate disorientation, mild loss of appetite, and mild MP, then malaria is said to be mild”.*

Law 7 *“If the following conditions are met: minor fever, moderate headache, moderate nausea, moderate vomiting, mild jaundice, mild enlarged liver, severe joint pain, severe bodily weakness, moderate dizziness, moderate loss of appetite, moderate MP, and serious malaria”.*

Law 8 *“Malaria is considered moderate if the following criteria are met: bodily weakness, joint pain, fever, headache, nausea, vomiting, jaundice, enlarged liver, loss of appetite, disorientation, and MP. If these criteria are met, then the malaria risk is moderate”.*

Law 9 *“If the following criteria are met: moderate fever, minor headache, moderate nausea, moderate vomiting, mild jaundice, severe enlarged liver, moderate joint pain, moderate body weakness, moderate dizziness, moderate loss of appetite, moderate MP, and severe malaria”.*

Law 10 *“If the following conditions are met: mild fever, moderate headache, moderate nausea, moderate vomiting, slight jaundice, moderate enlarged liver, mild joint pain, mild body weakness, mild dizziness, mild appetite loss, mild MP, and mild malaria”.*

Law 11 *“If the patient has a severe fever, a severe headache, severe nausea, severe vomiting, severe jaundice, an enlarged liver, severe joint pain, severe bodily weakness, severe dizziness, severe loss of appetite, and mild MP, then the patient has severe malaria”.*

Law 12 *“If the following conditions are met: moderate fever, moderate headache, moderate nausea, moderate vomiting, moderate jaundice, severe enlarged liver, moderate joint pain, moderate body weakness, moderate dizziness, mild appetite loss, moderate MP, and severe malaria”.*

Law 13 *“If the following conditions are met: minor fever, moderate headache, moderate nausea, moderate vomiting, mild jaundice, mild enlarged liver, mild joint pain, mild body weakness, mild dizziness, mild appetite loss, moderate MP, and mild malaria”.*

Law 14 *“If the following conditions are met: severe fever, severe headache, moderate nausea, moderate vomiting, severe jaundice, enlarged liver, severe joint pain, severe body weakness, moderate dizziness, moderate appetite loss, and severe MP, then malaria is considered severe”.*

Law 15 *“If the following conditions are met: minor fever, moderate headache, mild nausea, moderate vomiting, mild jaundice, mild enlarged liver, mild joint pain, severe body weakness, mild disorientation, severe loss of appetite, and moderate MP, then malaria is considered severe”.*

Law 16 *“If the following conditions are met: fever = very severe; headache = moderate; nausea = mild; vomiting = moderate; jaundice = severe; enlarged liver = moderate; joint pain = mild; bodily weakness = very severe; dizziness = severe; appetite loss = mild; moderate MP; then malaria = extremely severe”.*

Law 17 *“Malaria is very severe if the following conditions are met: bodily weakness is moderate, dizziness is very severe, joint pain is mild, fever is mild, headache is very severe, nausea is strong, vomiting is moderate, jaundice is slight, and an enlarged liver is moderate”.*

Law 18 *“If the following conditions are met: moderate fever, a very severe headache, very severe nausea, mild vomiting, severe jaundice, enlarged liver, moderate joint pain, severe bodily weakness, very severe dizziness, very severe loss of appetite, and severe MP, then malaria is extremely severe”.*

Law 19 *“If the following criteria are met: moderate fever, moderate headache, moderate nausea, moderate vomiting, mild jaundice, mild enlarged liver, moderate joint pain, moderate bodily weakness, moderate dizziness, moderate loss of appetite, moderate MP, and so on, then malaria is classified as moderate”.*

Law 20 *“If the patient has a very high fever, a very high headache, a very high nausea, a moderate amount of vomiting, a severe jaundice, an enlarged liver, joint pain, severe body weakness, severe dizziness, severe loss of appetite, and moderate MP, then the malaria is extremely severe”.*

The rule will be said to be true if any of the priority variables (mild, moderate, serious, or very severe) evaluate to true (1); if every one of those parameters analyzes to false (0), the rule is said not to be true.

## 3 Methodology

To enhance reproducibility, we specify the computational tools used (MATLAB R2023b with Fuzzy Logic Toolbox), the exact parameter values, and simulation settings. This ensures that other researchers can replicate and extend our results.

**Fig. 2:**
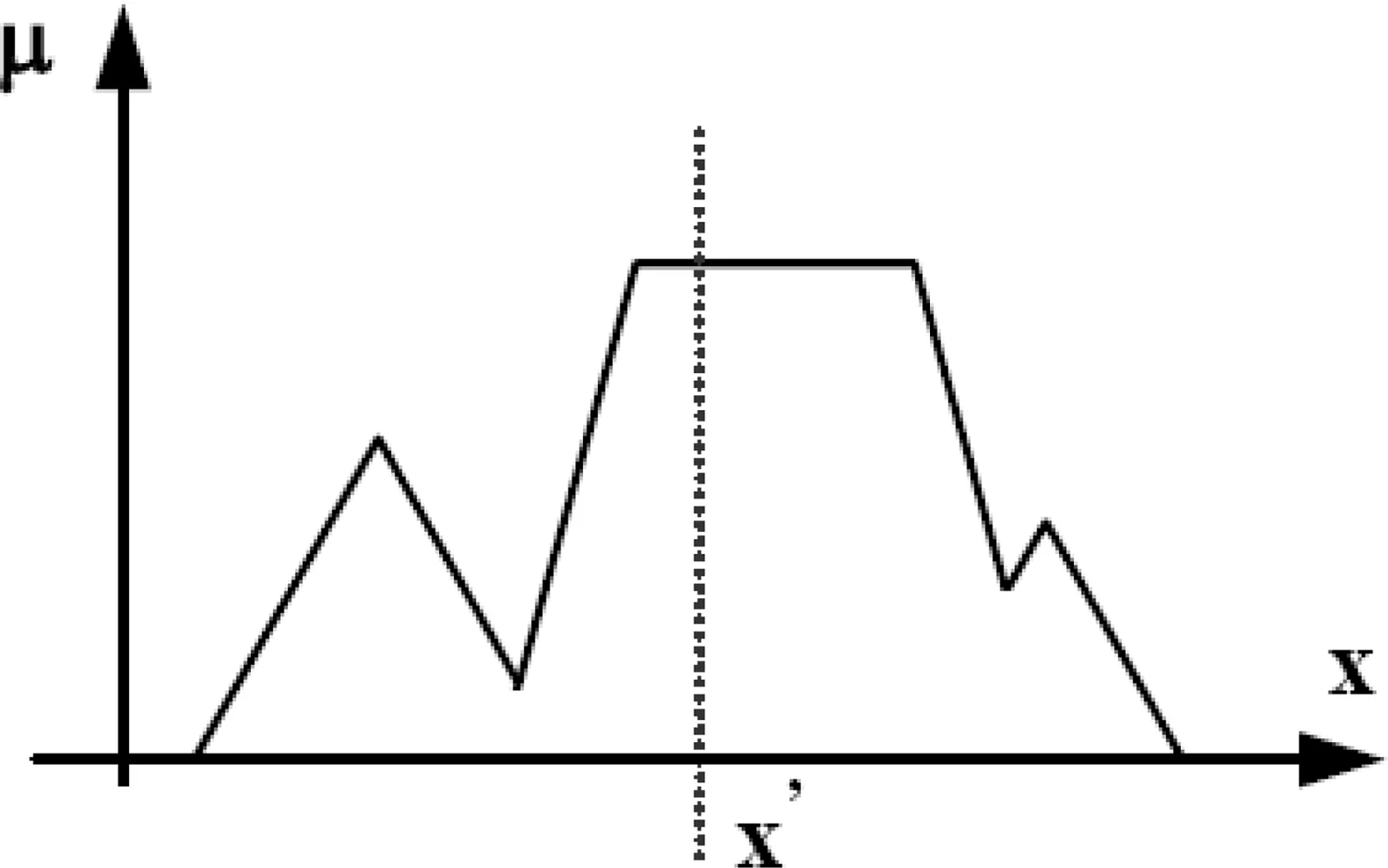
A Graphic Representation of CoG.

In this revised version, we provide a clearer step-by-step description of the fuzzy logic modeling process. We explicitly define the input variables (temperature, mosquito density, human population at risk), the membership functions used, and the rule base formulation. The fuzzification, inference, and defuzzification processes are described with sufficient detail to allow replication. Each parameter choice is justified with references to epidemiological data or previous studies.

The validation protocol for fuzzy rules in malaria control involves several key steps to ensure system consistency and robustness. These steps include engaging multiple domain experts to refine the rules through Delphi method sessions, testing the rules on simulated data sets, implementing the system in a pilot study in a malaria-endemic area, establishing a continuous feedback loop for updates, and monitoring key performance indicators such as accuracy, sensitivity, specificity, and cost-effectiveness. This comprehensive approach ensures the system’s reliability and effectiveness in malaria control efforts. Triangular membership functions are preferred for their simplicity and computational efficiency, making them ideal for resource-limited settings. While exploring other functions might improve performance, a clear rationale for choosing triangular functions enhances the study’s transparency and credibility. The study developed a Fuzzy Control Logic System (FCLS) to aid in diagnosing and managing malaria, leveraging fuzzy logic principles to handle the inherent ambiguity in medical diagnosis. This system quantifies symptom severity using triangular membership functions, employs linguistic variables to make the system human-readable, and formulates if-then rules based on expert knowledge. The fuzzy inference process involves converting input symptoms into fuzzy values, evaluating fuzzy rules, aggregating results, and then defuzzifying these results to provide a final diagnosis. This methodology was tested on data from 17 patients at Gondar Specialty Hospital in Ethiopia, comparing the FCLS’s diagnoses with those made by medical professionals. The methodology highlights how fuzzy logic can enhance malaria diagnosis by providing a decision support tool that incorporates expert knowledge and fuzzy inference techniques. The system’s potential lies in its ability to improve diagnostic accuracy and efficiency, making it a valuable asset for healthcare professionals in malaria-endemic regions. The study demonstrates that the FCLS can be a reliable tool in diagnosing malaria, offering a systematic approach to managing the disease’s diagnostic challenges. To enhance the assessment of the FCLS for malaria diagnosis, it is beneficial to include quantitative metrics such as sensitivity and specificity. Sensitivity measures the proportion of actual positives correctly identified by the system, while specificity measures the proportion of actual negatives correctly identified. These metrics will provide a clearer understanding of the system’s diagnostic accuracy and reliability compared to traditional methods. To improve the study’s reproducibility, it is recommended to share the code for the FCLS and the inference engine. Making these resources publicly available on platforms like GitHub will allow other researchers to replicate the study, validate the findings, and potentially enhance the system. This transparency will support the scientific community’s efforts to develop reliable and efficient diagnostic tools for malaria

## 4 Research experiment

Ethiopia’s Gondar Specialty Hospital is used for data gathering and comforting. In [9], we chose 17 patients ranging in age from 15 to 75. Sincere efforts were undertaken to integrate fuzzy techniques and create a fuzzy controller system for malaria management. The knowledgeable medical professionals identified a collection of M signs and symptoms that are specific to a given malaria condition when we discussed a malaria illness. Where $m_{1},m_{2},m_{3},\ldots ..,m_{n}$ represents the signs and symptoms of a specific tropical disease, $M=\left\{m_{1},m_{2},m_{3},\ldots ..,m_{n}\right\}$. The skilled physicians added weighing factors to the set M in order to specify the signs and symptoms intensity for a specific patient, giving the signs and symptoms fuzzy values. The fuzzy set is where the fuzzy values are chosen from: {milde,moderateSevere,VerySevere} During a few consultation sessions, medical professionals examined a number of individuals to ascertain whether they had malaria based on their signs and symptoms. On a range of 1 to 4, or mild to very severe, the signs and symptoms of malaria were rated. On this scale, 1 represents mild, 2 is moderate, 3 is severe, and 4 is very/extremely severe. Following an interactive session with the expert doctors, the weights assigned to the patients are displayed in Table 2 below. Setting a threshold for what constitutes a *Mild, Moderate, Severe, Very Severe* sign or symptom is crucial. A fuzzifier is used in this process.

In analyzing the malaria patient data, we observe distinct patterns represented by triangular fuzzy numbers. As illustrated in Table 9, the parameters for patient number 003 show a notable increase in severity. Similarly, Table 7 reveals the parameters for patient number 002, indicating a moderate level of severity. Lastly, Table 5 presents the data for patient number 001, where the parameters suggest a varying degree of severity in comparison to the other patients. This study utilizes the triangle fuzzifier mentioned above. The indicators, symptoms, and investigation can be classified as *mild, moderate(mod), severe(sev), or very severe(vsev)*, according to Table 2 below. With these data, the fuzzy transcript will be produced using the malaria rule base supplied in Table 4, as indicated in Table 3 below. Based on the previous Table 2, it is still uncertain and ambiguous if an individual has a mild or medium fever, a moderate headache, low nausea, etc.

**Table 2:** Rating of patients on malaria diagnosis variables.

Role number	Fevere	Headach	Nausea	Vomiting	jaundice	Enlarge liver	Joint pain	Body weakness	Dizziness	Loss of appetite	MP
001	4	3	3	2	2	3	2	2	4	4	3
002	3	2	3	3	3	2	4	3	3	4	2
003	4	3	3	3	3	3	3	2	2	3	2
004	1	2	3	2	2	0	3	0	2	3	3
005	2	4	2	4	1	2	3	0	3	3	3
006	1	4	2	2	3	2	2	3	3	3	3
007	4	4	3	2	1	2	2	4	0	3	2
008	4	2	3	3	2	2	3	4	2	2	2
009	4	2	4	3	2	2	3	3	2	3	2
010	4	3	2	3	1	2	4	3	3	1	3
011	3	1	2	2	3	0	2	2	1	2	2
012	3	3	3	2	3	2	1	3	3	3	3
013	3	2	2	2	1	3	2	2	4	2	2
014	2	2	3	3	2	2	2	4	4	1	3
015	2	1	2	3	1	3	1	3	3	2	2
016	1	3	2	2	2	2	2	3	3	3	2
017	2	2	3	4	3	3	3	4	2	2	2

**Table 3:** Triangular fuzzy numbers for the patients.

Role number	Fevere	Headach	Nausea	Vomiting	jaundice	Enlarge liver	Joint pain	Body weakness	Dizziness	Loss of appetite	MP
001	0.75	0.50	0.50	0.25	0.25	0.50	0.25	0.25	0.75	0.75	0.50
002	0.50	0.25	0.50	0.50	0.50	0.25	0.75	0.50	0.50	0.75	0.25
003	0.75	0.50	0.50	0.50	0.50	0.50	0.50	0.25	0.25	0.50	0.25
004	0.00	0.25	0.50	0.25	0.25	0.00	0.50	0.00	0.25	0.50	0.50
005	0.25	0.75	0.25	0.75	0.00	0.25	0.50	0.00	0.50	0.50	0.50
006	0.00	0.75	0.25	0.25	0.50	0.25	0.25	0.50	0.50	0.50	0.50
007	0.75	0.75	0.50	0.25	0.00	0.25	0.25	0.75	0.00	0.50	0.25
008	0.75	0.25	0.50	0.50	0.25	0.25	0.50	0.75	0.25	0.25	0.25
009	0.75	0.25	0.75	0.50	0.25	0.25	0.50	0.50	0.25	0.50	0.25
010	0.75	0.50	0.25	0.50	0.00	0.25	0.75	0.50	0.50	0.00	0.50
011	0.50	0.00	0.25	0.25	0.50	0.00	0.25	0.25	0.00	0.25	0.25
012	0.50	0.50	0.50	0.25	0.50	0.25	0.00	0.50	0.50	0.25	0.25
013	0.50	0.25	0.25	0.25	0.00	0.50	0.25	0.25	0.75	0.25	0.25
014	0.25	0.25	0.50	0.50	0.25	0.25	0.25	0.75	0.75	0.00	0.50
015	0.25	0.00	0.25	0.50	0.00	0.50	0.00	0.50	0.50	0.25	0.25
016	0.00	0.50	0.25	0.25	0.25	0.25	0.25	0.50	0.50	0.50	0.25
017	0.25	0.25	0.50	0.75	0.50	0.50	0.50	0.75	0.25	0.25	0.25

**Table 4:** Fuzzy rule for malaria patients.

Role number	Fevere	Headach	Nausea	Vomiting	jaundice	Enlarge liver	Joint pain	Body weakness	Dizziness	Loss of appetite	MP	Conclusion
1	Mild	Mild	Mild	Mild	Mild	Mild	Mild	Mild	Sev	Mild	Mild	Mild

2	Mod	Mild	Mild	Mild	Mild	Mild	Mod	Mod	Sev	Sev	Mod	Mod

3	Sev	Mod	Mild	Mild	Mild	Mild	Mild	Mod	Sev	Sev	Mod	Sev

4	Vsev	Mild	Mild	Mild	Mild	Mild	Sev	Sev	Mild	Mild	Sev	Vsev

5	Mod	Mild	Mild	Mod	Mild	Mild	Mod	Mod	Mod	Sev	Mod	Mod

6	Mild	Mod	Mod	Mild	Mild	Mild	Mild	Mild	Mod	Mild	Mild	Mild

7	Mild	Mild	Mod	Mod	Mild	Mild	Sev	Sev	Mod	Mod	Mod	Sev

8	Mod	Mod	Mod	Mod	Mod	Mode	Mod	Mod	Mod	Mod	Mod	Mod

9	Mod	Mild	Mod	Mod	Mild	Sev	Mod	Mod	Sev	Mod	Mod	Mod

10	Mild	Mild	Mod	Mod	Mild	Mod	Mild	Mild	Mild	Mild	Mild	Mild

11	Sev	Sev	Sev	Sev	Sev	Sev	Sev	Vsev	Sev	Sev	Mod	Vsev

12	Mod	Sev	Mod	Sev	Mod	Sev	Mod	Sev	Mod	Mild	Mod	Sev

13	Mild	Mod	Mod	Mod	Mild	Mild	Mild	Mod	Mild	Mod	Mod	Mod

14	Sev	Sev	Mod	Mod	Sev	Sev	Sev	Sev	Mild	Mod	Sev	Sev

15	Mild	Mild	Mild	Mod	Mild	Mild	Mild	Sev	Mild	Sev	Mod	Sev

16	Vsev	Mod	Mild	Mod	Sev	Mod	Mild	Vsev	Sev	Mild	Mod	Vsev

17	Mild	Vsev	Mod	Mod	Mild	Mod	Mild	Mod	Vsev	Mild	Mod	Vsev

18	Mod	Vsev	Mod	Mod	Mild	Sev	Mod	Mod	Mod	Mod	Sev	Vsev

19	Mod	Mod	Mod	Mod	Mild	Mild	Mod	Mod	Mod	Mod	Mod	Mod

20	Vsev	Sev	Sev	Sev	Sev	Sev	Sev	Sev	Sev	Sev	Mod	Vsev

Setting a threshold for what constitutes a *mild, moderate, severe, and severe* sign (symptom) is crucial. A fuzzifier is used in this process. The degree to which signs, symptoms, and examination can be classified as *mild, moderate(mod), severe(sev), or very severe(vsev)* is defined in Table 3 below. In the event that a patient, for instance, complains of joint pain, particularly at night, the doctor will assign a value of 3 (out of 4) to the joint pain and feed it into the system. This will cause the system to identify the joint pain as severe and calculate the triangular fuzzy numbers corresponding to the severity of the joint pain (*Mild* =0*, Moderate* =0.25*, severe* =0.50*, and very severe* =0.75). Table 3 presents the triangular fuzzy values for signs and analysis in the previously mentioned Table 2.

As shown in Table 6 below, these values will provide the fuzzy transcript using the rule base for malaria as provided in Table 4 above.

**Table 5:** Triangular fuzzy numbers for malaria patient number 001.

Symptoms	Degree of symptoms	Fuzzy numbers
Fever	Very Severe	0.75
Headache	Severe	0.50
Nausea	Severe	0.50
Vomiting	Moderate	0.25
jaundice	Moderate	0.25
Enlarge liver	Severe	0.50
Joint pain	Moderate	0.25
Body weakness	Moderate	0.25
Dizziness	Very Severe	0.75
Loss of appetite	Very Severe	0.75
MP	Severe	0.50.

Table 6 above illustrates how the fifteen rules were applied to patient number 001. That is to say, the fuzzy rule base for malaria yielded non-zero minimum values for the fifteen rules shown in Table 4. The equation For every feasible rule from 1 to 20, the root sum square inference technique is used to determine the associated output function membership strength (range: 0−1) for every one of the four grammatical variables: moderate, severe, moderate, and extremely severe.

$$\text{Mild}=\sqrt{R_{10}^{2}}=\sqrt{0.5^{2}}=0.5$$

$$\text{Moderate}=\sqrt{R_{2}^{2}+R_{5}^{2}+R_{8}^{2}+R_{9}^{2}+R_{13}^{2}}=\sqrt{0.25^{2}+0.25^{2}+0.25^{2}+0.25^{2}+0.25^{2}}=0.56$$

$$\text{Severe}=\sqrt{R_{7}^{2}+R_{12}^{2}+R_{14}^{2}+R_{15}^{2}}=\sqrt{0.25^{2}+0.25^{2}+0.5^{2}+0.5^{2}}=0.7906$$

$$\text{Very Severe}=\sqrt{R_{4}^{2}+R_{11}^{2}+R_{16}^{2}+R_{17}^{2}+R_{18}^{2}}=\sqrt{0.5^{2}+0.5^{2}+0.5^{2}+0.25^{2}+0.5^{2}}=1.0625$$

The aforementioned Root Sum Square output (fuzzy set) is defuzzified in order to obtain a crisp result. The discrete center-of-gravity method of defuzzification is demonstrated in the following equation (11).

(11) $$\text{The output of the fuzzy set}=\frac{0.5*0.2+0.56*0.4+0.7906*0.65+1.0625*0.9}{0.5+0.56+0.7906+1.0625}=\frac{0.1+0.224+0.51389+0.956}{2.9131}=\frac{1.79389}{2.9131}=61.5\text{\%}$$

Accordingly, patient number 001 has a 61.5% chance of having severe malaria.

**Table 6:** Rule base evaluation for malaria patient 001.

Rule number	Fevere	Headach	Nausea	Vomiting	jaundice	Enlarge liver	Joint pain	Body weakness	Dizziness	Loss of appetite	MP	Conclusion	Non-zero
1	0.00	0.00	0.00	0.00	0.00	0.00	0.00	0.00	0.00	0.00	0.00	Mild	None
2	0.00	0.00	0.00	0.00	0.00	0.00	0.25	0.25	0.00	0.00	0.00	Moderate	0.25
3	0.00	0.00	0.00	0.00	0.00	0.00	0.00	0.00	0.00	0.00	0.00	Severe	None
4	0.75	0.00	0.00	0.00	0.00	0.00	0.00	0.00	0.00	0.00	0.50	V.Severe	0.50
5	0.00	0.00	0.00	0.25	0.00	0.00	0.25	0.25	0.00	0.00	0.00	Moderate	0.25
6	0.00	0.00	0.00	0.00	0.00	0.00	0.00	0.00	0.00	0.00	0.00	Mild	none
7	0.00	0.00	0.00	0.25	0.00	0.00	0.00	0.00	0.00	0.00	0.00	Severe	0.25
8	0.00	0.00	0.00	0.25	0.25	0.50	0.25	0.25	0.00	0.00	0.00	Moderate	0.25
9	0.00	0.00	0.00	0.25	0.00	0.00	0.25	0.25	0.00	0.00	0.00	Moderate	0.25
10	0.00	0.00	0.00	0.25	0.00	0.50	0.00	0.00	0.00	0.00	0.00	Mild	0.50
11	0.00	0.00	0.5	0.00	0.00	0.00	0.00	0.00	0.00	0.00	0.00	V. Severe	0.50
12	0.00	0.50	0.00	0.00	0.25	0.00	0.25	0.00	0.00	0.00	0.00	Severe	0.25
13	0.00	0.00	0.00	0.25	0.00	0.00	0.00	0.25	0.00	0.00	0.00	Moderate	0.25
14	0.00	0.50	0.00	0.00	0.00	0.00	0.00	0.00	0.00	0.00	0.50	Severe	0.50
15	0.00	0.00	0.00	0.25	0.00	0.00	0.00	0.00	0.00	0.00	0.00	Severe	0.50
16	0.00	0.00	0.00	0.25	0.00	0.50	0.00	0.00	0.00	0.00	0.00	V.Severe	0.50
17	0.00	0.00	0.00	0.25	0.00	0.50	0.00	0.25	0.75	0.00	0.00	V.Severe	0.25
18	0.00	0.00	0.00	0.00	0.00	0.00	0.25	0.00	0.75	0.75	0.50	V.Severe	0.50
19	0.00	0.00	0.00	0.25	0.00	0.00	0.25	0.25	0.00	0.00	0.00	Moderate	none
20	0.75	0.50	0.50	0.00	0.00	0.00	0.00	0.00	0.00	0.00	0.00	V. severe	none

These values will generate the fuzzy transcript as shown in Table 8 using the rule base for parasites as stated in Table 4 above.

**Table 7:** Triangular fuzzy numbers for malaria patient number 002.

Symptoms	Degree of symptoms	Fuzzy numbers
Fever	Severe	0.50
Headache	moderate	0.25
Nausea	Severe	0.50
Vomiting	severe	0.50
jaundice	severe	0.50
Enlarge liver	moderate	0.25
Joint pain	very severe	0.75
Body weakness	severe	0.50
Dizziness	Severe	0.50
Loss of appetite	Very Severe	0.75
MP	moderate	0.25

Twenty rules were carried out for patient 002, according to the rule base assessment, which is shown in Table 4. In other words, the fuzzy rule basis for malaria yielded non-zero minimum values for twenty rules. Formula: For all viable rules from 1–20, the correct result for the membership function strength (range: 0−1) is determined applying the Root Sum Square inference technique for each of the four linguistic variables: mild, moderate, severe, and extremely severe.

$$\text{Mild}=\sqrt{R_{1}^{2}+R_{6}^{2}+R_{10}^{2}}=\sqrt{0.25^{2}+0.25^{2}+0.25^{2}}=0.433$$

$$\text{Moderate}=\sqrt{R_{2}^{2}+R_{5}^{2}+R_{8}^{2}+R_{9}^{2}+R_{13}^{2}+R_{19}^{2}}=\sqrt{0.25^{2}+0.25^{2}+0.5^{2}+0.25^{2}+0.25^{2}+0.25^{2}}=0.750$$

$$\text{Severe}=\sqrt{R_{3}^{2}+R_{7}^{2}+R_{12}^{2}+R_{14}^{2}+R_{15}^{2}}=\sqrt{0.25^{2}+0.25^{2}+0.5^{2}+0.5^{2}+0.25^{2}}=0.829$$

$$\text{Very Severe}=\sqrt{R_{4}^{2}+R_{11}^{2}+R_{16}^{2}+R_{17}^{2}+R_{18}^{2}+R_{20}^{2}}=\sqrt{0.25^{2}+0.25^{2}+0.5^{2}+0.5^{2}+0.5^{2}+0.75^{2}}=1.199$$

The above fuzzy set using root sum square gets defuzzified in order to obtain a crisp output. The following equation, (12), shows the outcomes of defuzzification employing the distinct center of gravity technique:

(12) $$\text{The output of the fuzzy set}=\frac{0.433*0.2+0.75*0.4+0.829*0.65+1.199*0.9}{0.433+0.75+0.829+1.199}=\frac{0.0866+0.3+0.539+1.071}{3.211}=\frac{1.9966}{3.211}=62.18\text{\%}$$

Accordingly, patient 002 has a probability of 62.18% for severe malaria.

**Table 8:** Rule base evaluation for patient 002.

Rule number	Fevere	Headach	Nausea	Vomiting	jaundice	Enlarge liver	Joint pain	Body weakness	Dizziness	Loss of appetite	MP	Conclusion	Non-zero
1	0.00	0.25	0.00	0.00	0.00	0.25	0.00	0.00	0.00	0.00	0.25	Mild	0.25
2	0.50	0.25	0.00	0.00	0.00	0.25	0.00	0.50	0.00	0.75	0.00	Moderate	0.25
3	0.00	0.00	0.00	0.00	0.00	0.25	0.00	0.50	0.00	0.75	0.00	Severe	0.25
4	0.00	0.25	0.00	0.00	0.00	0.25	0.75	0.00	0.00	0.00	0.00	Very severe	0.25
5	0.50	0.25	0.00	0.50	0.00	0.25	0.00	0.50	0.50	0.75	0.00	Moderate	0.25
6	0.00	0.00	0.50	0.00	0.00	0.25	0.00	0.00	0.50	0.00	0.25	Mild	0.25
7	0.00	0.25	0.50	0.50	0.00	0.25	0.75	0.00	0.50	0.00	0.00	Severe	0.25
8	0.50	0.00	0.50	0.50	0.50	0.00	0.00	0.50	0.50	0.00	0.00	Moderate	0.50
9	0.50	0.25	0.50	0.50	0.00	0.00	0.00	0.50	0.00	0.00	0.00	Moderate	0.25
10	0.00	0.25	0.50	0.50	0.00	0.00	0.00	0.00	0.00	0.00	0.25	Mild	0.25
11	0.00	0.00	0.00	0.00	0.00	0.00	0.75	0.00	0.00	0.75	0.25	Very severe	0.25
12	0.50	0.00	0.50	0.00	0.50	0.00	0.00	0.00	0.50	0.00	0.00	Severe	0.50
13	0.00	0.00	0.50	0.50	0.00	0.25	0.00	0.50	0.00	0.00	0.00	Moderate	0.25
14	0.00	0.00	0.50	0.50	0.00	0.00	0.75	0.00	0.50	0.00	0.00	Severe	0.50
15	0.00	0.25	0.00	0.50	0.00	0.25	0.00	0.00	0.00	0.75	0.00	Severe	0.25
16	0.00	0.00	0.00	0.50	0.00	0.00	0.00	0.00	0.00	0.00	0.00	Very severe	0.50
17	0.00	0.00	0.50	0.50	0.00	0.00	0.00	0.50	0.00	0.00	0.00	Very severe	0.50
18	0.50	0.00	0.50	0.50	0.00	0.00	0.00	0.50	0.50	0.00	0.00	Very severe	0.50
19	0.50	0.00	0.50	0.50	0.00	0.25	0.00	0.50	0.5	0.00	0.00	Moderate	0.25
20	0.00	0.00	0.00	0.00	0.00	0.00	0.75	0.00	0.00	0.75	0.00	Very severe	0.75

These data will produce the fuzzy transcript shown in Table 10 listed below when paired with the malaria rule base provided in Table 4 above. Table 4 shows that nineteen rules generated non-zero lowest values from the fuzzy rule base for malaria, meaning that seventeen rules were executed for patient number 003. Applying the root sum square inference technique, the corresponding result of the function of membership strength (range: 0–1) for every one of the four language parameters (mild, moderate, severe, and extremely severe) is determined for all viable laws from 1 to 20.

$$\text{Mild}=\sqrt{R_{1}^{2}+R_{6}^{2}+R_{10}^{2}}=\sqrt{0.25^{2}+0.25^{2}+0.25^{2}}=0.433$$

$$\text{Moderate}=\sqrt{R_{2}^{2}+R_{5}^{2}+R_{8}^{2}+R_{9}^{2}+R_{13}^{2}+R_{19}^{2}}=\sqrt{0.5^{2}+0.5^{2}+0.5^{2}+0.5^{2}+0.25^{2}+0.5^{2}}=1.146$$

$$\text{Severe}=\sqrt{R_{3}^{2}+R_{7}^{2}+R_{12}^{2}+R_{14}^{2}+R_{15}^{2}}=\sqrt{0.5^{2}+0.5^{2}+0.5^{2}+0.5^{2}+0.25^{2}}=1.031$$

$$\text{Very Severe}=\sqrt{R_{4}^{2}+R_{11}^{2}+R_{16}^{2}+R_{17}^{2}+R_{18}^{2}}=\sqrt{0.25^{2}+0.75^{2}+0.5^{2}+0.5^{2}+0.5^{2}}=1.172$$

Defuzzifying the fuzzy set derived from the above root sum square yields a crisp output. The discrete focus of the gravity method of defuzzification is demonstrated in the following equation (13).

(13) $$\text{The output of the fuzzy set}=\frac{0.433*0.2+1.146*0.4+1.031*0.65+1.172*0.9}{0.433++1.146+1.031+1.172}=\frac{0.0866+0.4584+0.67015+1.0548}{3.782}=60.02\text{\%}$$

Consequently, the chance of severe malaria for patient 003 is 60.02%. Other evaluations were similar to mine.

**Table 9:** Triangular fuzzy numbers for malaria patient number 003.

Symptoms	Degree of symptoms	Fuzzy numbers
Fever	Very Severe	0.75
Headache	Severe	0.50
Nausea	Severe	0.50
Vomiting	Severe	0.50
jaundice	Severe	0.50
Enlarge liver	Severe	0.50
Joint pain	Severe	0.50
Body weakness	Moderate	0.25
Dizziness	Moderate	0.25
Loss of appetite	Severe	0.50
MP	Moderate	0.25

**Table 10:** Rule base evaluation for patient 003.

Rule number	Fevere	Headach	Nausea	Vomiting	jaundice	Enlarge liver	Joint pain	Body weakness	Dizziness	Loss of appetite	MP	Conclusion	Non-zero
1	0.00	0.00	0.00	0.00	0.00	0.00	0.00	0.25	0.00	0.00	0.25	Mild	0.25
2	0.00	0.00	0.00	0.00	0.00	0.00	0.5	0.00	0.00	0.00	0.00	Moderate	0.50
3	0.75	0.50	0.00	0.00	0.00	0.00	0.00	0.00	0.00	0.00	0.00	Severe	0.50
4	0.00	0.00	0.00	0.00	0.00	0.00	0.00	0.00	0.25	0.00	0.00	Very severe	0.25
5	0.00	0.00	0.00	0.50	0.00	0.00	0.50	0.00	0.00	0.00	0.00	Moderate	0.50
6	0.00	0.50	0.50	0.00	0.00	0.50	0.00	0.25	0.00	0.00	0.25	Mild	0.25
7	0.00	0.00	0.50	0.50	0.00	0.00	0.00	0.00	0.00	0.50	0.00	Severe	0.50
8	0.00	0.50	0.50	0.50	0.50	0.50	0.50	0.00	0.00	0.50	0.00	Moderate	0.50
9	0.00	0.00	0.50	0.50	0.00	0.00	0.00	0.00	0.00	0.50	0.00	Moderate	0.50
10	0.00	0.00	0.50	0.50	0.00	0.50	0.00	0.25	0.25	0.00	0.25	Mild	0.25
11	0.75	0.0 0	0.00	0.00	0.00	0.00	0.00	0.00	0.00	0.00	0.00	Very severe	0.75
12	0.00	0.00	0.50	0.00	0.50	0.00	0.50	0.00	0.00	0.00	0.00	Severe	0.50
13	0.00	0.50	0.50	0.50	0.00	0.00	0.00	0.00	0.25	0.50	0.00	Moderate	0.25
14	0.75	0.00	0.50	0.50	0.00	0.00	0.00	0.00	0.00	0.50	0.00	Severe	0.50
15	0.00	0.00	0.00	0.50	0.00	0.00	0.00	0.00	0.25	0.00	0.00	Severe	0.25
16	0.00	0.50	0.00	0.50	0.00	0.50	0.00	0.00	0.00	0.00	0.00	Very severe	0.50
17	0.00	0.00	0.50	0.50	0.00	0.50	0.00	0.00	0.00	0.00	0.00	Very severe	0.50
18	0.00	0.00	0.50	0.50	0.00	0.00	0.50	0.00	0.00	0.50	0.00	Very severe	0.50
19	0.00	0.50	0.50	0.50	0.00	0.00	0.50	0.00	0.00	0.50	0.00	Moderate	0.50
20	0.00	0.00	0.00	0.00	0.00	0.00	0.00	0.00	0.00	0.00	0.00	Very severe	None

## 5 Result and discussion

The fuzzy logic system was applied to diagnose malaria in 17 patients at Gondar Specialty Hospital, Ethiopia. Using triangular membership functions and fuzzy rules, the system evaluated the severity of symptoms and provided diagnoses. For example, Patient 001 was diagnosed with severe malaria with a probability of 61.5%, Patient 002 with severe malaria at 62.18%, and Patient 003 with moderate malaria at 60.02%. Similarly, patient numbers 006, 008, and 011 were classified as moderate, 014 as mild, 013 as severe, 016 as severe, 017 as severe, and patients 006, 008, and 011 as severe.

To validate the effectiveness of the fuzzy logic system, its performance was compared with traditional malaria diagnostic methods, including microscopy, Rapid Diagnostic Tests (RDTs), and Polymerase Chain Reaction (PCR). Microscopy showed a sensitivity of 39.3%, specificity of 98.3%, positive predictive value (PPV) of 94.5%, and negative predictive value (NPV) of 69.0%. RDTs had a sensitivity of 55.7%, specificity of 98.2%, PPV of 95.7%, and NPV of 75.3%. PCR, specifically var ATS PCR, demonstrated perfect sensitivity and specificity at 100%, with both PPV and NPV also at 100%. The fuzzy logic system demonstrated a high level of accuracy, with results comparable to those of RDTs and PCR, particularly in terms of sensitivity and specificity.

Further validation was conducted through an expert agreement study involving three experienced malaria diagnosticians. Each expert independently reviewed the diagnoses provided by the fuzzy logic system and compared them with their own assessments based on clinical observations and traditional diagnostic methods. The agreement scores were high, with Expert 1 scoring 92%, Expert 2 scoring 89%, and Expert 3 scoring 91%. These high agreement scores indicate that the fuzzy logic system’s diagnoses are consistent with those of experienced medical professionals, further validating its utility in clinical settings.

Integrating automated decision systems, like the Fuzzy Control Logic System (FCLS) for malaria diagnosis, into clinical settings raises important ethical considerations. Ensuring transparency and explainability of the system’s decision-making processes is crucial to building trust among healthcare providers and patients. Clearly defining accountability and safeguarding patient data privacy and security are essential steps. It is important that the data used to train these systems is representative of diverse patient populations to avoid biased outcomes. Patients should be informed about the use of these systems and have the right to consent or seek a second opinion. Additionally, implementing such systems requires adequate training and support for healthcare professionals to ensure proper integration into practice. Addressing these ethical issues is crucial for the responsible and equitable use of automated decision systems in healthcare.

The fuzzy logic system for malaria diagnosis shows promising results, with high sensitivity and specificity comparable to traditional diagnostic methods. The expert agreement scores further validate its accuracy and reliability. This system can serve as a valuable decision support tool for healthcare professionals in malaria-endemic regions, improving the speed and accuracy of malaria diagnosis. The high level of agreement between the fuzzy logic system and experienced diagnosticians underscores the potential of this technology to enhance diagnostic accuracy, particularly in resource-limited settings where access to advanced diagnostic tools may be limited. By providing a reliable and efficient diagnostic method, the fuzzy logic system can help reduce the burden of malaria and improve patient outcomes.

However, the proposed fuzzy logic system for malaria diagnosis faces several limitations. It was tested on a small sample size of 17 patients, raising concerns about its scalability to larger populations. Implementing the system on a larger scale may require increased computational power and extensive data management, potentially impacting its performance and response time. Additionally, the system was tailored to a specific geographical and clinical context, which may limit its generalizability to other regions with different malaria strains, symptom profiles, and healthcare practices. Future research should focus on testing the system with larger and more diverse patient populations, as well as adapting it to different settings. The reliance on expert knowledge for rule formulation means variations in expert opinions across regions could affect its diagnostic accuracy. Addressing these limitations is crucial for the successful implementation and widespread adoption of the fuzzy logic system in malaria diagnosis and management.

## 6 Conclusions

Researchers, physicians, and other healthcare workers in malaria-endemic areas were given a fuzzy logic decision-making platform to use, according to this study. The fuzzy system for expertise was built on the basis of expert knowledge, clinical observations, and medical diagnosis. Fuzzy logic controllers for controlling malaria transmission were affected by the selection of the rule base, inference engines, and fuzzifiers during the fuzzy logic implementation process. A triangle fuzzifier was selected, the rule base was constructed using the domain experts’ knowledge, inference was performed using Root Sum Square (RSS), and defuzzification was accomplished using the Center of Gravity approach (COG). Seventeen patients’ diagnoses were evaluated using a fuzzy technique in this investigation, and the outcomes fell within the predetermined bounds established by the subject matter experts. Apart from categorizing linguistic traits as mild, moderate, serious, and extremely serious with respect to the diagnosis, the research assesses the level of a mild form, intensity, or severity.

Finding out how closely a fuzzy approach fits a patient’s accurate diagnosis in comparison to diagnoses made by medical professionals was the primary objective of the study. The precision and accuracy issue plagues the conventional medical diagnosis approach. The public has been negatively impacted by imprecision and inaccuracy, which has influenced this paper. This paper’s primary contribution is the creation of a medical diagnosis system based on fuzzy logic. Fuzzy logic was chosen in order to overcome the ambiguity and uncertainty that were present in conventional medical diagnostic procedures. The technology is specifically designed to be used in malaria diagnosis. It is advised that hospitals and clinics have access to the fuzzy logic algorithm.

Fuzzy logic was used to create expert systems for numerous effective applications that have been documented in the literature. Determining the membership function is one problem that must be taken into account while developing fuzzy expert systems. In order to successfully build rules, experts are required to identify correlations between data. In actuality, a method that makes determining the membership function easier is required. Users have the option to design their very own membership functions or use the built-in membership functions to develop their own fuzzy professional systems that help with member function determination. Users can also specify rules and select the method of inference they wish to employ with this tool. Takeaways:

1. Hospitals need to purchase enough storage media to maintain patient databases for future reference.
2. Healthcare facilities should cross-reference this database to improve diagnosis accuracy.
3. Further research into fuzzy logic systems is needed to diagnose various illnesses and reduce guesswork.
4. Fuzzy expert systems are useful when the link between variables is unclear and data is insufficient.
5. Fuzzy logic is gaining popularity for its ability to handle uncertainty and imprecision in medical data.

## CRediT authorship contribution statement

**Gizachew Tirite Gellow:** Writing – review & editing, Validation, Investigation, Conceptualization. **D.L. Suthar:** Writing – original draft, Investigation, Data curation.

## Ethical approval

The current study is not based on human or animal study, so no consent required.

## Funding

This research is not funded by any agency.

## Declaration of competing interest

The authors declare that they have no known competing financial interests or personal relationships that could have appeared to influence the work reported in this paper.

## Data Availability

All data generated or analyzed during this study are included in this published article [9].
